# Mechanical Properties of Epoxy Networks with Metal Coordination Bonds: Insights from Temperature and Molar Mass Variation

**DOI:** 10.1021/acs.macromol.4c01143

**Published:** 2024-09-16

**Authors:** Benke Li, Stelios Alexandris, Christos Pantazidis, Esmaeel Moghimi, Georgios Sakellariou, Dimitris Vlassopoulos, Emmanouela Filippidi

**Affiliations:** † Institute of Electronic Structure and Laser, FORTH, Heraklion, 70013, Greece; ‡ Department of Chemistry, National and Kapodistrian University of Athens, Athens, 15784, Greece; § Department of Materials Science and Engineering, University of Crete, Heraklion, 70013, Greece

## Abstract

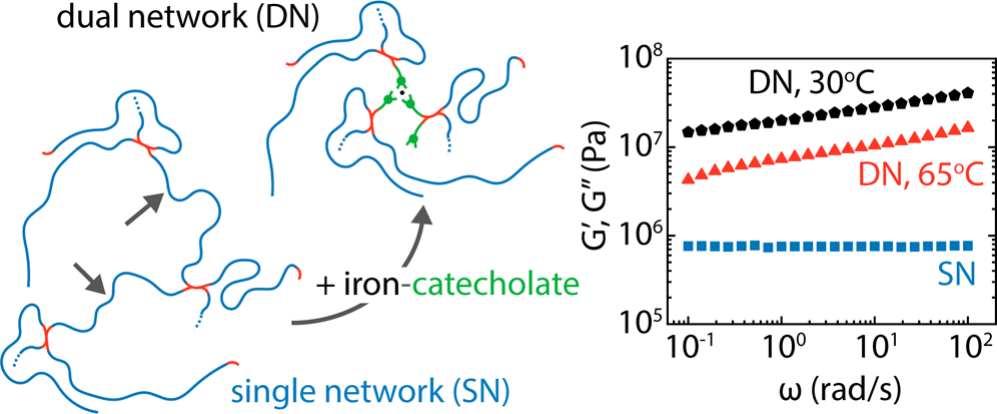

We investigate the thermal and mechanical properties of poly(ethylene glycol), PEG, networks with either solely covalent epoxy bonds (single networks, SNs) or coexisting epoxy and iron–catecholate bonds (dual networks, DNs). The latter has recently been shown to be a promising material that combines mechanical strength with significant deformability. Here, we address the previously unexplored effects of the temperature and PEG precursor molar mass on the mechanical properties of the networks. We focus on PEG molar masses of 500 g/mol, where crystallization is suppressed, and 1000 g/mol, where some weak crystals are formed. SNs soften with an increasing PEG molar mass. Heating reversibly softens the DN, but it has a minimal effect on SNs. Nonlinear shear deformation of the DN breaks iron–catecholate bonds, and subsequent recovery upon shear cessation occurs to a long-time steady-state modulus whose value is almost triple the original one, likely due to the formation of tris-complexes versus initial sterically or kinetically trapped bis-complexation. The response under elongation indicates that the DN with sacrificial bonds is stiffer and more extensible than the other networks. These results may provide guidelines for designing dual networks with tunable mechanics at the molecular level.

## 1 Introduction

Developing high-modulus unfilled elastomers that exhibit exceptional stiffness, strength, and toughness poses a significant challenge. These qualities are highly desirable in applications where high extensibility and damage tolerance are paramount. However, achieving this combination of properties has proven to be anything but straightforward.^1−6^ Creative strategies to control the microscopic architectures such as mechanical interlocking,^2^ use of double or triple interpenetrating polymer networks,^3−5^ mixing metal coordination,^6,7^ or hydrogen bonds^8,9^ along with covalent bonds have all been successful in creating macroscopically resilient materials. In previous works,^6,10^ we developed a synthetic, rehealing, dry elastomer with two types of bonds: a minimal level of covalent bonds ensuring network connectivity and iron–catecholate coordinate bonds inspired by marine mussels to add stiffness to the network. While the exquisite resulting mechanical properties were attributed to the cooperative effects of the dual cross-linking strategies, a number of formidable challenges await to be addressed pertaining to the degree of mechanical property enhancement depending on the polymer of choice, the sustainability of the networks, and the deeper understanding of the mechanical relevance of iron–catecholate networks in nature.

Within this research landscape, a crucial question is the role of the macromolecular precursor in forming the epoxy network. The use of poly(ethylene glycol) (PEG) combines features such as water-based chemistry, biocompatibility and pharmaceutical relevance, numerous telechelic functionalities, and—a focal point of this work—a variety of molar masses, enabling the comparative study of the use of shorter amorphous and longer semicrystalline chains on the properties of the dually bonded networks.

To examine the link between the molecular features and the macroscopic mechanical response, we employ shear and extensional rheology. Whereas the former provides information on the shear modulus, linear viscoelastic response, and a hint about the network’s self-healing behavior, the latter probes the resistance to tension and maximum extensibility of the network. We compare the epoxy elastomeric single networks (SN) formed solely by a chemical cross-linker and PEG of different molar mass to a dual network (DN) containing both chemical and iron–catecholate bonds and present a systematic set of experiments involving the kinetics of cross-linking, thermal properties, and rheological and mechanical properties. We thus provide an improved understanding of the precursor network connectivity and isolate behaviors pertaining only to the DNs. Such knowledge will be used for the future development and molecular design of such composite macromolecular materials.

## 2 Materials and Methods

### 2.1 Network Formation

Telechelic epoxide functionalized poly(ethylene glycol) diglycidyl ether (PEGDE-500, CAS 26403-72-5) with number-average molar mass *M*_n_ = 500 g/mol, tetrafunctional 1,4-diaminobutane (DAB, CAS 110-60-1) of 88.15 g/mol, iron(III) nitrate nonahydrate (CAS 7782-61-8), and bicine (CAS 150-25-4) were purchased from Sigma-Aldrich. PEGDE-1000 with *M*_n_ = 1000 g/mol was purchased from PolySciences Inc. Triethylsilane-protected eugenol was synthesized following the method of Seo et al.^11^ An epoxide functionality was added following the method of Heo et al.^12^ We refer to the final liquid product as “protected catechol” (CAT) (394.69 g/mol). NMR spectra of the synthesized moieties are shown in Figure S1. Single epoxy networks were synthesized per composition in Table 1 by mixing PEGDE and DAB stoichiometrically in terms of functional groups. As one PEGDE has two functional groups (epoxides) and a DAB has two primary amines that can react four times in total, stoichiometry requires 2 PEGDE for every 1 DAB. We prepare two types of networks, based either on PEGDE-500 or on PEGDE-1000. We refer to PEGDE-500/DAB networks as SN-500 and PEGDE-100/DAB networks as SN-1000. Both PEGDE (500 and 1000 g/mol) and DAB were brought to their liquid state well before being mixed together. Similarly, protected catechol networks were created by mixing PEGDE, DAB, and CAT stoichiometrically in terms of functional groups. We refer to the DAB-CAT-PEGDE-500 networks as CAT-500, and those with PEGDE-1000 as CAT-1000. A couple of drops of tetrahydrofuran were added as a compatibilizer. For the rheological response in time, the mixture was stirred at 60 °C. For a large-area film creation, the mixture was vortexed and degassed for 10 min, at <1 Torr pressure. Monomer mixtures were poured and cured between PTFE slides with a 0.5 mm spacer at 60 °C and 600 Torr for over 24 h. The chosen mixing ratio in CAT networks ensured the formation of minimally covalently cross-linked networks.^13^

**Table 1:** Mixing Compositions and Glass Transition Temperatures (*T*_g_) of Polymer Networks

network	% composition	polymer (PEGDE)	DAB cross-linker	CAT protected catechol	*T*_g_ (°C)
SN	mole ratio PEGDE:DAB = 1.0:0.5				
SN-500	mass%	91.9	8.1	—	–40.6
SN-1000	mass%	95.78	4.22	—	–50.2
CAT	mole ratio PEGDE:DAB:CAT = 1.00:0.885:1.54				
CAT-500	mass%	42.164	6.579	51.257	–15.9
CAT-1000	mass%	59.318	4.627	36.055	–48.5

**Figure 1:**
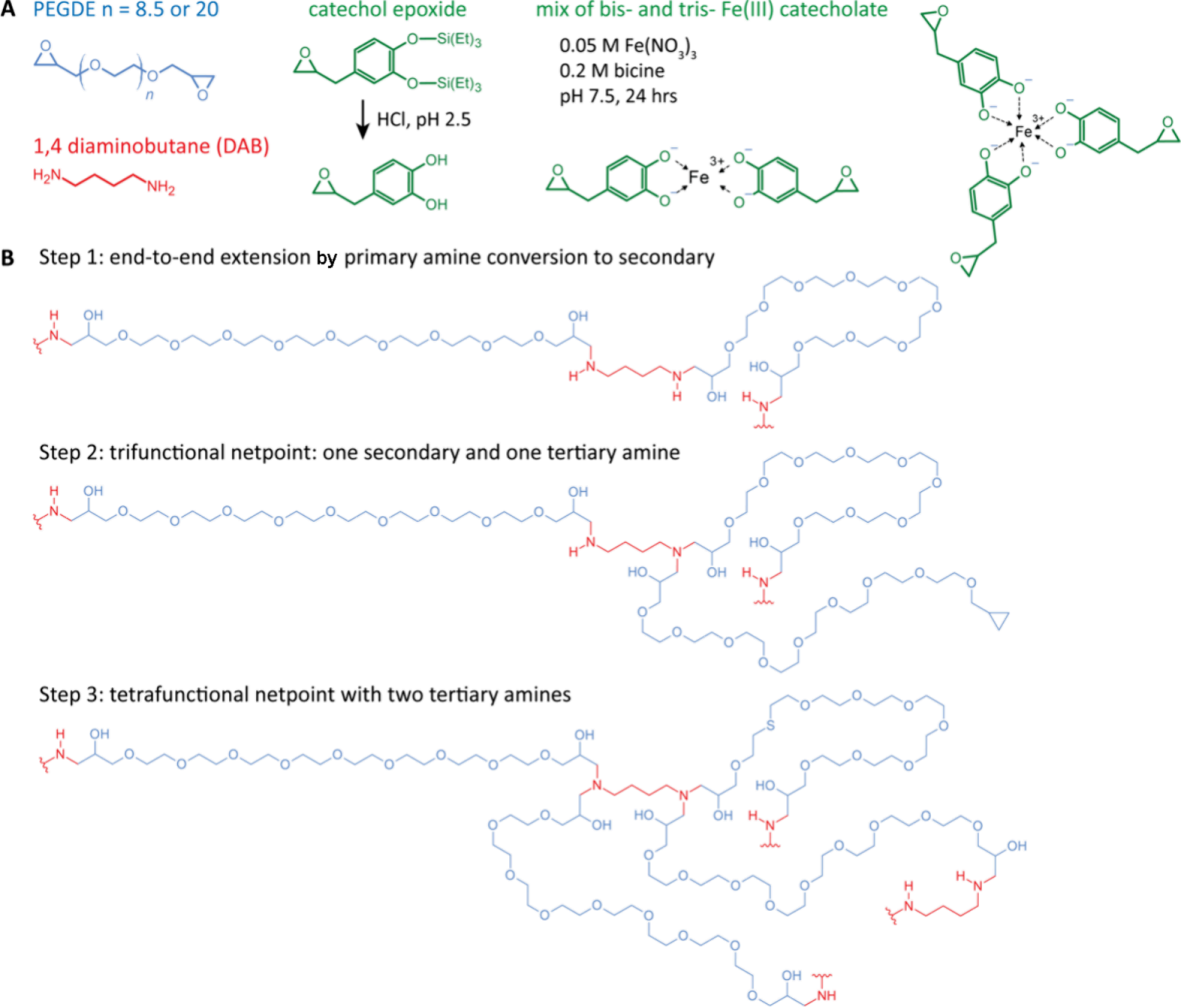
(A) Schematic illustration of telechelic PEGDE precursors of *M*_n_ 500 g/mol and 1000 g/mol corresponding to 8.5 and 20 monomers and 3.6 and 7.3 Kuhn segments,^21^ respectively; the tetrafunctional amine cross-linker DAB; the protected and deprotected catechol epoxide; a bis-iron-catecholate complex formed at pH 7.5, which is the most prevalent expected form in dilute stoichiometric solutions^22^ compared to the tris- complexes formed at higher pH, above 9.1. Previous studies^6^ have shown a mix of bis- and tris-coordination in our system. (B) The reaction steps leading to SN formation (adapted with permission #5856881124017; Copyright 2017, *Science*, AAAS).

Specimens were cut in disks for shear rheology or in stripes for extensional rheology. SNs were used as is without swelling and redrying. CAT network specimens were subjected to 200-fold excess solution (per mass) compared to the mass of the samples degassed with HCl at pH 2.5 overnight at room temperature to enforce triethylsilyl protecting group deprotection. Homogeneous iron introduction was achieved by transferring the already swollen specimens to a solution of 0.05 M Fe(NO_3_)_3_·9H_2_O and 0.2 M bicine in ∼10-fold excess of iron compared to the tris-stoichiometric ratio of 1 Fe:3 catechols, titrated to pH 7.5 with NaOH (measured by pH meter) for 24 h. Typical photographs of the samples under treatment are shown in Figure S7. After treatment, swollen specimens were gently positioned on a net for a few minutes at ambient conditions where fast evaporation ensued, followed by drying in a vacuum oven at 50 Torr and 30 °C for at least 48 h. Any noncomplexed iron that has entered the network is thus not removed. A schematic illustration of the network components is presented in Figure 1A. For comparing the effect of pH, iron introduction at a pH of 4.0, 7.7, and 10.53 was done. It is worth noting that treatment at or above a pH of 11.5 is impossible, as iron precipitates from the solution.

### 2.2 Infrared Spectroscopy (IR)

Transmission IR spectra during cross-linking at 60 °C were obtained at the Lambda 950 UV/vis spectrometer (PerkinElmer) at the wavelength range of 1500–2500 nm (or equivalently 6666–4000 cm^–1^) and steps of 1 nm.

### 2.3 Differential Scanning Calorimetry (DSC)

DSC (TA Instruments DSC 250) measurements were initialized with an isothermal step at 110 °C for 2 min to remove humidity, followed by two full cooling–heating cycles in the range of −100 to 80 °C with a heating and cooling rate of 10 °C/min. The resulting scans of the two cycles were identical. The results of the second cooling cycle are shown in Figure 3. The inflection points of the cooling traces were used for the determination of the glass transition temperatures, *T*_g_ (Table 1, Figure 3).

**Figure 2:**
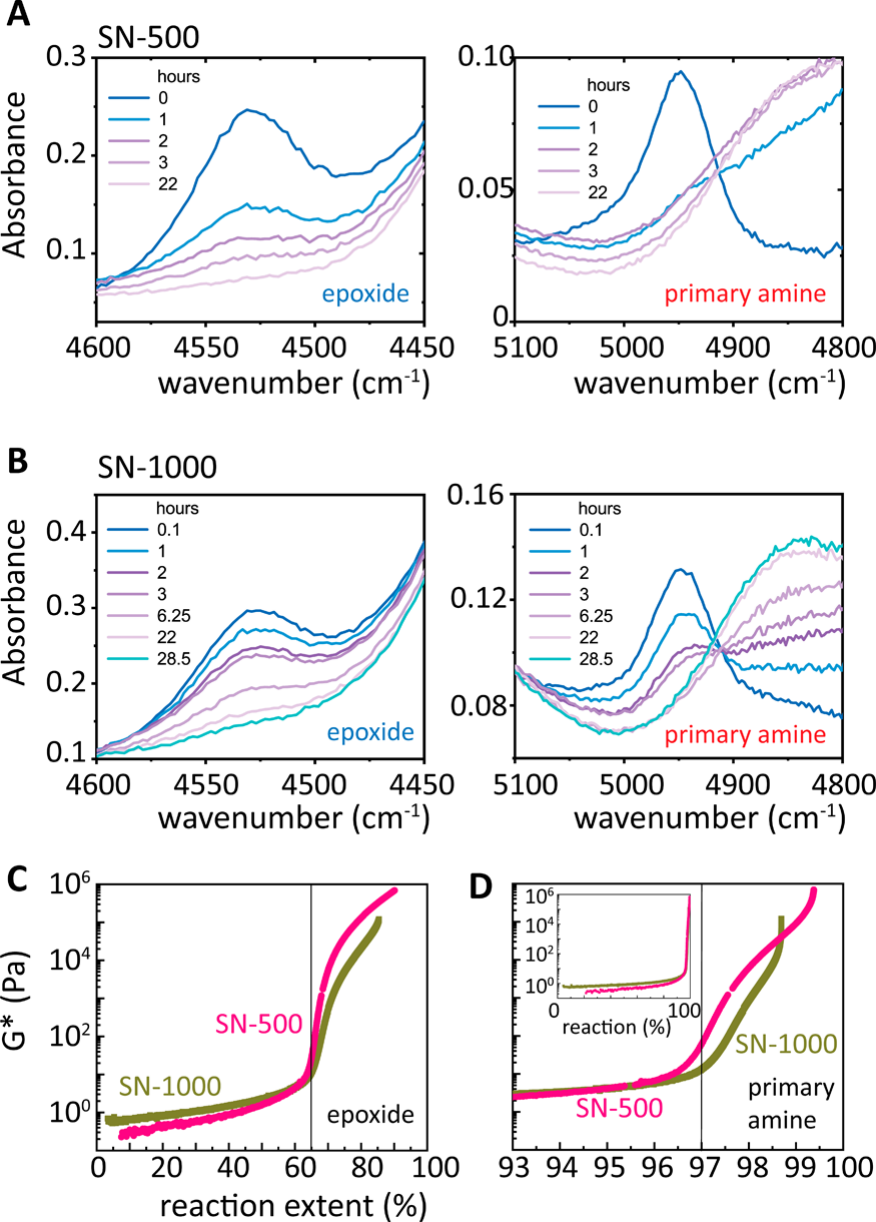
Absorbance IR spectra for the consumption of the epoxide (A) and primary amine (B) over time. (C, D) Isothermal evolution at 60 °C of the complex modulus, G*, versus the reaction extent for epoxide and primary amine, for SN-500 (C) versus SN-1000 (D). Vertical guides to the eye are at *X*_*n*_^epoxide^ = 65% for A and *X*_*n*_^amine^ = 97% for B. Inset in (D) shows G* as a function of reaction extent.

**Figure 3:**
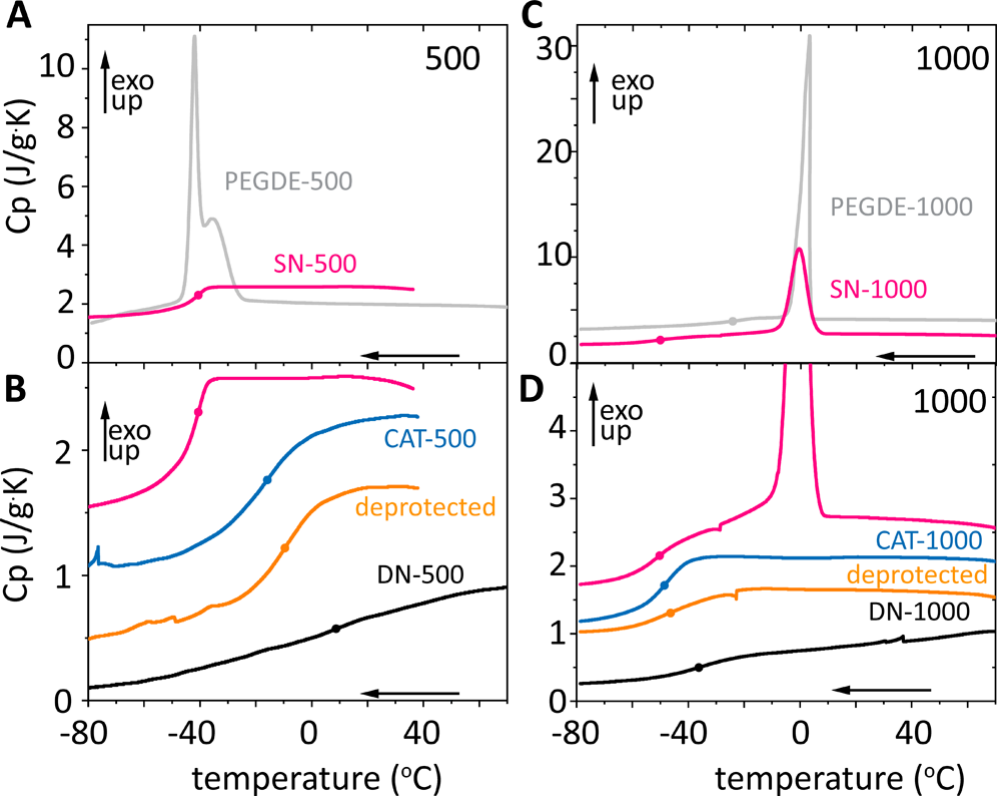
Differential scanning calorimetry (cooling) of the PEG500 (A, B) and PEG1k (C, D) melts and networks. Melt (gray), single networks (pink), CAT-networks with protected (blue) and deprotected (orange) catechol, and iron-treated networks (black). Circles mark the fitted glass transition temperatures. Curves are shifted vertically for clarity.

### 2.4 Rheology

The cross-linking reaction was monitored at 60 °C (same temperature as standard curing) by dynamic time sweep tests using an MCR 702 rotational rheometer (Anton Paar, Austria) operating in the strain-control mode, with nitrogen gas flux, a stainless steel parallel-plate geometry (diameter 25 mm and about 1 mm gap), a probing frequency of 10 rad/s, and initial strain amplitude of 5%, reduced to 0.1% after the moduli rose appreciably. Linear and nonlinear shear measurements of the elastomeric networks were performed with an ARES 2kFRTN1 strain-controlled rotational rheometer (TA, USA) with stainless steel parallel plates (4 mm diameter and about 0.45 mm gap) coated with sandpaper (320 grit) to reduce the risk of wall slip. Special care was taken to ensure good adhesion of the plates by gently squeezing the sample and allowing the normal force to relax. Temperature control of ±0.1 °C was achieved with a convection oven fed with nitrogen gas to create an inert atmosphere. We performed three types of oscillatory measurements: (i) dynamic frequency sweeps at fixed linear strain amplitude γ = 0.1% and fixed temperatures (30, 40, and 65 °C) to probe the linear viscoelastic (LVE) spectrum; (ii) dynamic strain sweep at a fixed frequency ω = 10 rad/s to determine the limits of linear viscoelastic response and assess the nonlinear deformation of the networks, followed by (iii) a dynamic time sweep at a fixed frequency ω = 10 rad/s and linear strain amplitude γ = 0.1% to probe the kinetics of their structural recovery. For (i), frequency sweeps are reported at steady state with temperature-equilibrated samples.

For the extensional measurements we used the Sentmanat extension rheometer (SER) fixture^14−16^ mounted on an ARES rheometer. It consists of two counter-rotating drums, with the specimen fixed onto their outer surface with clamps. The measurements were performed at temperatures of 30 and 65 °C. The maximum Hencky strain achieved was just below 2 (Figure 5), while that corresponding to one full rotation of the drums is 4. Specimens were cut into approximately 17 mm long, 3–6 mm wide strips. The aspect ratio, defined as the width divided by the thickness, was always between 6 and 8. To prevent slippage of the sample on the drums, sandpaper was glued on the surface of the drums and the clamps, and a layer of tape was placed on top of the clamps. The experimental operation followed the procedure of Aho et al.^17^ The extension rate was set to *ε̇*_H_ = 0.01 s^–1^. This measurement provides information on the engineering stress, and the extension is nearly uniaxial.^18^

**Figure 4:**
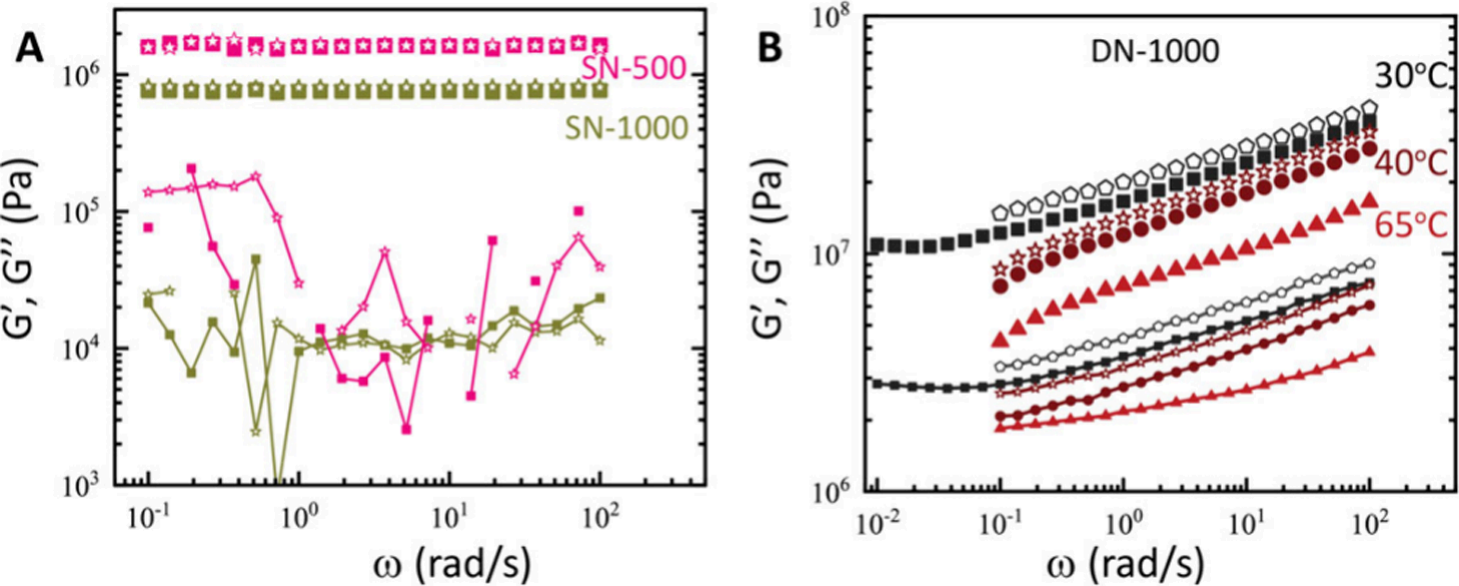
Frequency-dependent storage (*G′*, symbols) and loss (*G″*, line plus symbol) moduli at strain amplitude 0.1% for (A) SN-500 (pink) and SN-1000 (olive) at 30 °C (filled) and 65 °C (open) and for (B) DN-1000 at 30, 40, and 65 °C. In B the temperature was increased from 30 to 40 °C to 65 °C (filled symbols) and subsequently decreased back to 40 and 30 °C (open symbols).

**Figure 5:**
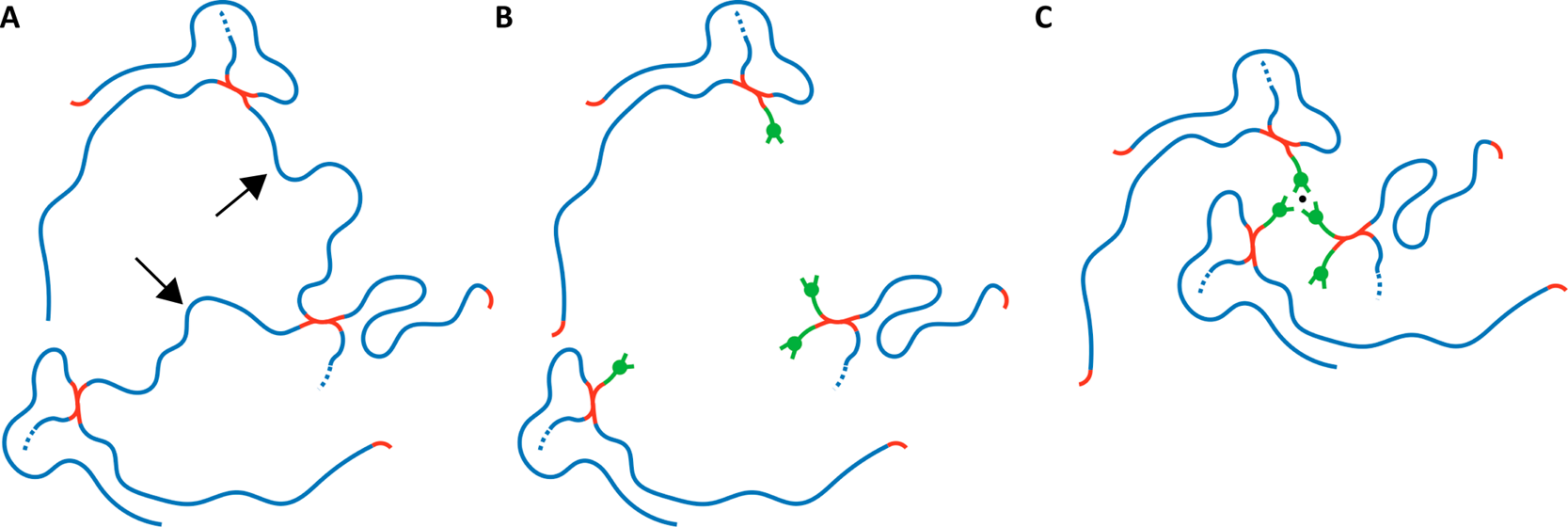
(A–C) Schematic illustration truthful to 2d chemical structure of the local architecture expected for the (A) SN-1000 network versus (C) DN-1000. (B) The network in (A) with the PEGDE chains marked by arrows replaced by catechols.

**Figure 6:**
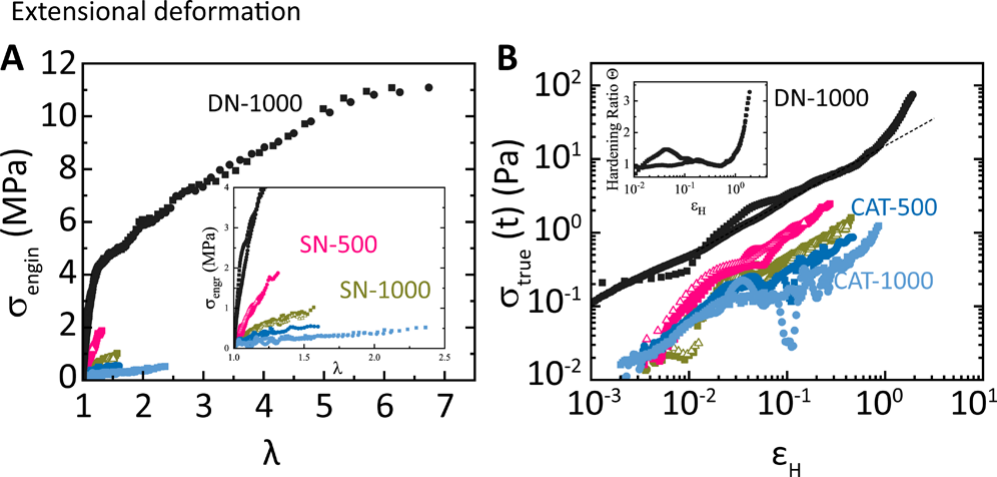
(A,B) Extensional rheometry results. (A) Engineering stress as a function of stretch ratio at 30 °C (filled symbols) and 65 °C (open symbols). Inset in (A) shows the data over a smaller range of λ. (B) True stress vs Hencky strain *ε*_H_ = ln λ. The dashed line is the extension of the linear region response *σ*_true__,LVE_(*t*). Inset in (A): strain hardening ratio, Θ = *σ*_true_(*t*)/*σ*_true__,LVE_(*t*). The same color code is used in both plots.

## 3 Results and Discussion

### 3.1 Curing Kinetics

Single network formation consists of the reaction of the oligomeric telechelic PEGDE with the tetrafunctional DAB via an epoxide-amine reaction. Curing kinetics were examined by IR^19^ at 60 °C, where the consumption extent of the epoxide (4530 cm^–1^) and the primary amine (4950 cm^–1^) was followed (Figures 1C,D, S2, and S3), and concurrent LVE moduli measurement. Similar studies of linking epoxy-amine kinetics to mechanics have been employed by using Raman in combination with Brillouin scattering.^20^ Near IR shows that the primary amine is being quickly consumed (∼1 h), and its consumption was significantly slower in the case of the 1000 g/mol PEGDE. Curing kinetics were also followed via shear rheology (Figure S2C,D). LVE moduli were recorded by means of dynamic time sweep tests at a probing frequency of 10 rad/s. Using different, e.g., 10-fold faster or slower, probing frequencies is not expected to have altered our observations, due to the *a posteriori* observation of frequency-independent *G′* in that regime (Figure 4A). An early increase in loss modulus (*G*″) coincident with a nonmeasurable storage modulus (*G′*) and a fast primary amine consumption point toward a fast end-to-end extension step (Figure 1B) where the viscosity increases due to the presence of longer, more massive, yet un-cross-linked structures. For a given DAB molecule to act as a netpoint between these now elongated linear chains, it requires it to have undergone three reactions (of the possible four), which should be equivalent to 75% epoxide consumption. At this stage, a concurrent increase in *G*′ and a crossover with *G*″ takes place (see Figure 2). A final fourth reaction, if possible, reinforces the network and increases the topological complexity. An epoxide consumption below 100% after 28 h points kinetically trapped epoxides. By fitting the reaction extents *X*_*n*_(*t*) of the primary amine and the epoxide over time, and having measured *G′*(*t*) and *G″*(*t*), we transform the results into G*(*X*_*n*_), i.e., the evolution of the complex modulus with respect to the epoxide or amine consumed during cross-linking (Figure 2). First, it becomes evident that the slowdown of reactions in the case of 1000 g/mol PEGDE in IR observations is purely kinetic, as G*(*X*_*n*_) is similar for both PEGDE molar masses. Second, as expected, almost complete consumption (∼97%) of the primary amine has taken place before significant development of network character (inset of Figure 2B). Third, the glass transition of the completely reacted network is over 100 °C lower (Figure 3B,D) than the curing temperature, so at no stage during the reaction should molecular mobility become severely restricted. Fourthly, for both PEGDE molar masses, the consumption of the epoxide is ∼65% at the time of a steep G* rise (about 4 orders of magnitude); this is a ∼10% smaller reaction extent than expected, pointing toward the effective linking of already elongated chains, thus giving rise to early network formation and possible inhomogeneities associated with the extent and distribution of network junctions. The weaker and eventually gradual increase of moduli at a larger reaction extent following the first steep rise reflects the increased degree of cross-linking. The question of how many, on average, molecules are participating in the end-to-end extension cannot be resolved by IR, and thus we resort to linear viscoelasticity to obtain additional insights.

### 3.2 Thermal Properties

Precursors and networks of the two oligomers were thermally characterized by DSC. Figure 3 presents the DSC cooling traces, wherein the crystallinity of the 1000 g/mol SN (SN-1000) is established, as opposed to the 500 g/mol SN (SN-500), where cross-linking is sufficient to suppress crystallinity (Figure 3A,C). The segments between cross-links created in the SN-1000 (see Section 3.3 below) are likely to crystallize as extended chains^23^ between netpoints, as opposed to folded chains, due to their low total, elongated molar mass. At 30 °C, the lowest temperature where mechanical measurements are performed, SN-1000 is sufficiently far from the crystallization temperature, but is still within the melting regime (peak *T*_m_ = 24 °C, see Figure S3), and characteristic PEO crystalline regions are evident in wide-angle X-ray scattering (Figure S4) superimposed on a prominent amorphous peak.

It is worth noting that for both molar masses CAT-networks are noncrystalline loose networks, as the bulky protected (blue) or deprotected (orange) CAT that replaces 45% mol of PEGDE is sufficient to suppress crystallinity (Figure 3B,D). Fe-treated networks (DN-networks) with complex architecture and topology exhibit a broad DSC trace, reflecting the superposition of overlapping local processes. In similar networks based on poly(propylene oxide) (PPO-380 and PPO-640) the similarly occurring broad DSC trace could not be individually resolved in either DSC or temperature-modulated DSC.^24^ Glass transition temperatures exhibit similar trends for protected, deprotected, and Fe-treated networks in both SN-500 and SN-1000, although their values are shifted by 30 °C for SN-1000 (Table 1).

### 3.3 Linear Viscoelastic Spectra

We performed dynamic frequency sweep measurements with the three types of networks (SN-, CAT-, and DN-networks that contain dual bonds, epoxy, and coordination) at both molar masses and at three temperatures, 30, 40, and 65 °C (Figures 4 and S3) The SN-500 data (Figure 4A) reflect strong elastomeric networks with virtually frequency-independent storage (*G′*) and loss (*G″*) moduli. Furthermore, they display no temperature dependence between 30 and 65 °C. For SN-1000, the value of *G*′ is 45% that of SN-500. The average value of the loss factor *G″*/*G′* for SN-500 is about 0.0056, whereas for SN-1000 it is 0.0125, in agreement with the expectation that the loss factor typically decreases with increasing cross-link density.^25^

According to X-ray scattering, no network heterogeneities are observed in the networks up to 1 nm length scales (Figure S4). The storage modulus can be rationalized in the context of network models. Given the molecular structure of the present networks (Figure 1), we consider the phantom network model where the cross-link junctions can fluctuate.^21^ The predicted plateau modulus , where *f* is the functionality of the cross-link junction, ρ the density, *R* the ideal gas constant, *T* the absolute temperature, and *M*_c_ the average molar mass of the segments between cross-links. By assuming *f* = 4 and using the experimentally obtained *G′*(30 °C) (Table 2) and ρ^500^ = 1.2 g/mL and ρ^1000^ = 1.1 g/mL, we extract *M*_c_^500^ = 850 g/mol and *M*_c_^1000^ = 1730 g/mol (*M*_c_^1000^:*M*_c_^500^ ≈ 2.0), which correspond to 1.4 and 1.6 PEGDE-DAB repeats, respectively. As the epoxide conversion is above 85% (Figures 2 and S2E,F), we can use recent models of network elasticity^26,27^ that account for the linear effect of the concentration of defects (loops, dangling ends, dispersity of network strands) on the network’s shear modulus. Following the calculations of Lin et al.^27^ for a bifunctional polymer (A_2_) reacting with a tetrafunctional cross-linker (B_4_, *f* = 4) and conversion *p* = 0.9, we obtain for dangling ends (eq 16 therein)  − 2(1 − *p*) + O((1 − *p*)^2^) = 0.5 − 0.2 + *O*(0.01) ≅ 0.3. Theoretical^28^ and experimental^29^ work on bifunctional polymers with trifunctional junctions for PEG polymers similar to our molar mass with ∼20 repeat units and cross-linking taking place at nearly the melt state points to primary and secondary loop fractions < 0.05. The resulting reduction in modulus , as read in Figure 5 of the work of Lin et al.^27^ Therefore, invoking the contribution of dangling ends arrives closer to the experimentally measured plateau modulus compared to the reduction due to the existence of loops. We thus conclude that dangling ends are the main contributor to deviation from the phantom model in our case. Using *M*_c_^500^ = 500 and *M*_*c*_^1000^ = 1000, the expected *G′* values are *G′*_phantom_^500^ = 3 MPa, *G′*_dangl_^500^ = 1.8 MPa, *G′*_phantom_^1000^ = 1.4 MPa, and *G′*_dangl_^1000^ = 0.8 MPa, in good agreement with theory.^27^

**Table 2:** Mechanical Properties of the Networks at 30 °C

Network	*E*_slope_ (MPa)	*E*_nH_^a^ (MPa)	*E*_Mooney__–Rivlin_ (MPa)	*U*_r_ (MPa)	*G*′ plateau (MPa)	λ_fracture_ – 1
SN-500	6.13	7.83	9.00	0.6 ± 0.3	1.78	0.31
SN-1000	2.21	2.49	3.27	0.9 ± 0.1	0.80	0.57
CAT-500	1.42	1.41	1.77	0.7 ± 0.2	0.42	0.60
CAT-1000	0.62	0.66	0.84	1.0 ± 0.2	0.21	1.36
DN-1000	41.5	37.8	36.6	131 ± 11	10.9 (0.01 s^–1^), 16.7 (1 s^–1^)	5.12

a nH = neo-Hookean.

It is worth noting that the DAB with MW = 88.15 g/mol with its linear chain of four carbon and two nitrogen atoms does not alter the flexibility of linearly connected chains, as opposed to stiff cross-linkers often used in industrial epoxy networks such as diethyl toluene diamine (DETDA), in which the amines are directly linked to the aromatic ring. We additionally note that the 588 g/mol chain in the ideal network between netpoints comprises only four Kuhn segments, resulting in an oligomer rather than a Gaussian chain.

The loosely cross-linked CAT-networks (Figure S5) are much softer, as expected, with plateau values (taken at the frequency corresponding to minimum tan(δ)) of *G′*_CAT__,500_ = 0.42 MPa and *G′*_CAT__,1000_ = 0.21 MPa. Given that the 45 mol % of protected catechols act as pendant groups to the DAB molecules, cross-link functionality on average is reduced from *f* = 4 to 3. For the phantom network, we find *M*_c_^CAT,500^ ≅ 2400 g/mol and *M*_c_^CAT,1000^ ≅ 4400 g/mol, corresponding to 4.1 and 4.0 (PEGDE-DAB) repeats or 18 and 32 Kuhn segments, respectively, numbers consistent with the loose network structure that we aimed to synthesize.

The Fe-treated dual network of 1000 g/mol PEGDE precursor (DN-1000) studied with shear rheology (Figure 4B) exhibits at 30 °C and 1 rad/s an elastic modulus *G′*_DN__,1000_ = 16.7 MPa, an 80-fold increase from the CAT-1000 network and 21-fold increase from the SN-1000. That clearly showcases the remarkable mechanical performance rooted in the additional cross-linking coupled with the changes in network architecture and other cooperative effects taking place upon iron–catecholate coordination.^6^ As the temperature increases from 30 to 65 °C, *G′* drops. For example, at ω = 1 rad/s, *G*′ decreases from 16.7 MPa (at 30 °C) to 12.1 MPa (at 40 °C) and 7.50 MPa (at 65 °C), which cannot be explained by network elasticity (which suggests that the product *ρT* increases weakly), so should originate from the breakup of physical bonds. In this network, there is a mix of trifunctional iron–catecholate octahedra and tetrafunctional DAB; thus naively, 3 < *f* < 4. Whether considering *f* = 4 or 3 and the phantom model or the affine model, we obtain apparent *M*_c_ values that are unphysically small (maximum value for the affine model is 254 g/mol ≪1000 g/mol of the precursor PEGDE). This happens because we are neglecting an effect of foremost importance: iron coordination alters the topology of cross-linking by eliminating the need to have a polymer chain between two DAB cross-links (Figure 5A–C). In turn this creates an infinitely stiff local connection, with greatly reduced fluctuations. This stiff local connection depends on temperature, which affects the dissociation constant *K*_d_ of the tris-complexes and relaxes the strong binding. The altered topology compared to the CAT-networks is already visible at the time of iron-introduction, manifesting itself as a reduction in sample volume (Figure S7), and has been previously partially illustrated by experiments that modulated the catecholate fraction.^10^

To further assess the temperature dependence of the iron–catecholate bonds, we resort to studies of Fe(III)-tris(catechol) density functional theory (DFT) calculations^30^ as the best available estimates of the temperature dependence of the dissociation constant *K*_d_, being aware that our most probable complexation states are a mix of bis- and tris-complexes.^6^ Gas phase DFT confirms that the reaction is exothermic and spontaneous and predicts the Gibbs free energy |Δ*G*| and *K*_d_ as a function of temperature. There is a decreased spontaneity of the reaction as temperature rises. The probable loss of Fe–catecholate bonds is directly reflected in the decrease of *G′*. The agreement between gas phase DFT applied to our network (Table S2 and calculations thereof) and rheological data (Figure 4B) is reassuring.

Upon temperature reversal, *G′* recovers (open symbols). Actually, it slightly but consistently increases to a new steady state. For example, at ω = 1 rad/s and 30 °C, it rises from 16.7 MPa initially to 20.2 MPa after the full temperature cycle (Figure 4B). Shearing (even within the linear regime) coupled with temperature-induced bond breaking likely allows the system to explore different configurations and bond connectivities, which were previously kinetically trapped.

### 3.4 Extensional Deformation

The mechanical performance of the dual network is highlighted in the extensional testing performed at 30 and 65 °C (Figure 6A,B). Despite some scattering (due to measurement resolution and sample imperfections), data are well fit by the neo-Hookean and Mooney–Rivlin models.^31−33^ For the neo-Hookean model, the engineering stress is *σ*_eng_ = *G*(λ – 1/λ^2^), or the true stress is *σ*_true_ = *G*(λ^2^ – 1/λ), where *G* is the shear plateau modulus of the material with  for incompressible materials in elongation, *E* is the Young’s modulus, and λ = *l*/*l*_0_ = exp(*ε*_H_) is the stretch ratio (with *l*_0_ and *l* being the initial and final dimension).^21^ This model is suitable within the linear elastic response region (small deformations *ε*_H_ < 0.2 or λ < 1.22).^33^ For the Mooney–Rivlin model, *σ*_eng_ = (2*C*_1_ + 2*C*_2_/λ)(λ – 1/λ^2^) or *σ*_true_ = (2*C*_1_ + 2*C*_2_/λ)(λ^2^ – 1/λ), where *C*_1_ and *C*_2_ are empirically determined fit constants, and *G* = 2(*C*_1_ + *C*_2_). This model is valid until a weakly nonlinear elastic response region. Conforming to expectations, SN-1000 is less stiff (*E*^1000^ = 2.49 MPa) than SN-500 (*E*^500^ = 7.83 MPa) and deforms more until fracture, owing to its longer *M*_c_. This imparts a 47% higher modulus of resilience *U*_r_ = ∫ *σ*_eng_*dλ*, i.e., *U*_r_^1000^ = 0.93 MPa versus *U*_r_^500^ = 0.63 MPa. As seen in Figure 6A,B, a temperature increase from 30 to 65 °C has virtually no effect either on the SN-500 or on the SN-1000, in agreement with linear viscoelastic spectra. The values of moduli and deformation at break for the different samples investigated are listed in Table 2 (see also Figure S9).

Compared to SN, both the shear modulus and Young’s moduli of the CAT-networks drop sharply, with a concomitant increase of the maximum stretch ratio at fracture, *λ*_fracture_ – 1. As a result, the loose network has a slightly increased total resilience modulus. For the DN-1000, both the Young’s modulus and the strain at fracture increase by an order of magnitude each with respect to the SN-1000, from *E* = 2.49 MPa to 37.8 MPa and from *λ*_fracture_ – 1 = 0.57 to 5.12. Consequently, the modulus of resilience increases over 2 orders of magnitude, from *U*_r_ = 0.93 MPa to 131 MPa. This finding demonstrates the outstanding mechanical performance of these materials. Plotting the calculated true stress versus Hencky strain (Figure 6A) helps appreciate the material’s strain hardening behavior. The networks SN-500, SN-1000, and CAT-500 fracture at Hencky strain values *ε*_H_ < 0.5, following a nearly linear elongation (stress vs strain). However, for more extensible samples (CAT-1000 and DN-1000), fracture occurred at *ε*_H_ ≈ 1 to 2, following a strong strain hardening transition at *ε*_H_ ≈ 1. This strain hardening was attributed to stretching of the network strands.^15,21^ The hardening ratio (also called strain hardening factor), Θ = *σ*_true_(*t*)/*σ*_true__,LVE_(t), where *σ*_true__,LVE_ is the linear viscoelastic envelope,^18^ provides a metric of the tensile strength of the samples. For the Fe-treated DN-1000 sample, it increases from 1 to 3.3 (inset of Figure 6B). We hypothesize that breaking and reforming of iron–catecholate bonds is taking place: during extension, the shorter chains between chemical cross-links that are connected by iron–catecholate bonds fracture and rebond elsewhere. The process continues with the next longer chain segments and so on and so forth, further increasing the strain hardening ratio. The result is an enhanced extensibility under the action of tension until the covalent network eventually breaks.

### 3.5 Large-Amplitude Oscillation: Bond Breakup and Reversibility

**Figure 7:**
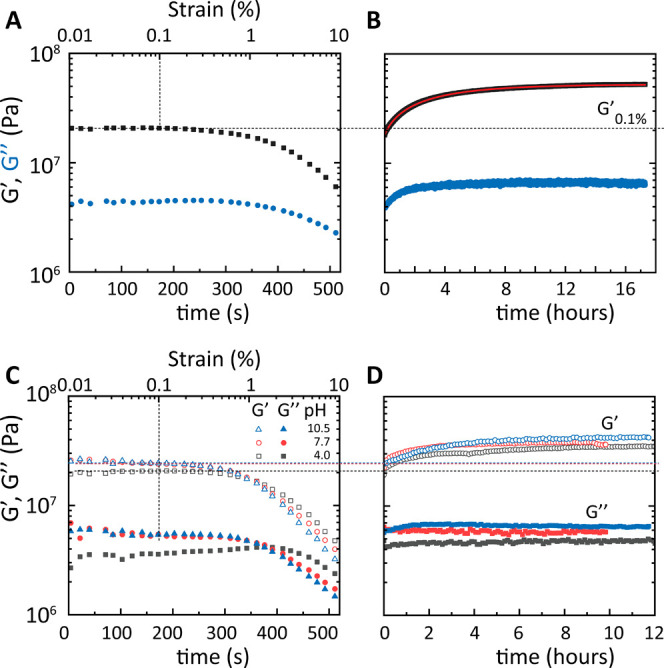
Strain sweep and recovery of the DN-1000 network at 30 °C. (A) Having previously erased the history at 65 °C, a dynamic shear strain sweep in the range 0.01–10% partially fractures the sample (breaks the Fe–catecholate bonds). (B) Time-dependent recovery at a frequency of ω = 1 rad/s and strain amplitude of γ = 0.1%. The dashed line shows an initial modulus value at strain 0.1%. Red line is the fit (see text). (C, D) Identical break and reversibility measurements for samples treated at lower and higher pH iron solutions. pH values higher than 7.5 appear to have minimal effect on the mechanics.

To assess the reversibility and self-healing of the dual network, we performed two consecutive tests: (i) dynamic strain sweep tests at 1 rad/s and 30 or 65 °C, spanning a wide strain amplitude range from 0.01% to 10%, well within the nonlinear regime, and (ii) dynamic time sweep at the same frequency and temperature and strain amplitude of 0.1% in the linear regime, to probe the recovery of the moduli (at 30 °C Figure 7 and at 65 °C Figure S10). We observe that upon increasing the strain amplitude, both moduli drop (as much as one decade in the present experiments), but the apparent *G′* remains higher than the apparent *G″* in the nonlinear regime because the network maintains its macroscopic coherence. This decrease marks the breaking of iron–catechol sacrificial bonds, thus softening the network.^34^ Upon cessation of the nonlinear deformation (having reached an amplitude of 10%), these bonds reform and the network’s structure rebuilds, as implied by the recovery of the storage modulus, depicted in the dynamic time sweep immediately following the strain sweep. Analysis of Figure 7B yields the network’s recovery time. It takes 8.9 min to recover the initial modulus *G*′_0.1%_. This is comparable (slightly faster) to the time needed for complete recovery of the Young’s modulus after a 10% tensile deformation for the DN-500.^6^ Notably, after complete recovery of the storage modulus, reaching the original plateau value, we observe that it continues to increase, reaching after 17 h a long-time steady-state value of *G′*_∞_ = 52.75 MPa, which is 2.6-fold higher than the initial modulus (*G′*_0.1%_ = 20.03 MPa). The time evolution of *G′*(*t*) in Figure 7B is fit by *G′*(*t*) = (*G′*_∞_ – *G′*_0_)[1 – e^–(*t*/*τ*_net_)β^ ] + *G*′_0_, where *G′*_0_ = 18.0 MPa is the initial modulus at the time sweep, *τ*_net_ = 3.41 h is the network’s rebuilding time, and the fitting parameter β = 0.88 is the stretch parameter. Similar findings are observed at 65 °C (Figure S11). The effect persists for samples treated at pH 4.0, 7.5, and 10.5, as shown in Figure 7C,D, pointing to an across-the-border underlying phenomenon. One potential explanation is the excess of iron present, arising from the over-stoichiometric iron introduction and the nonwashing of the iron at the end of the treatment. To rationalize this long recovery to a value of the storage modulus that is about triple its original value, we conjecture a “topology optimization of bonds”, i.e., the formation of tris-complexes versus initial bis-complexes during the process and breaking and reformation. By analogy, in filled polymers, weak flow has been shown to be an effective strategy to aid the system in overcoming the local minima of bond energy traps, thus enabling the system to reach its globally optimized structure.^35^ Future spectroscopy experiments will focus on confirming the presence of bond breaking and rearrangement and the mobility of metal coordination bonds in a dry polymeric environment. This phenomenon may provide new possibilities to explore the mechanics and microstructure evolution of dual stiff and highly stretchable yet self-healing networks that need to be further investigated.

## 4 Concluding Remarks

Single and dual PEG networks were prepared with either solely covalent epoxy bonds in the former case or both covalent and iron–catecholate coordination bonds. The PEG molar mass was chosen to vary by a factor of 2 (from 500 to 1000 g/mol), which impacted the weak crystallinity of the SN-1000 at 30 °C but did not impart any crystallinity in either the CAT- or derived networks. We followed the increase in network strength with the extent of reaction and performed shear and extensional rheological measurements. The results revealed softening of the SN with increasing molar mass of the PEG precursor. The description of the plateau modulus in the context of the phantom network model suggests that the networks are heterogeneous. The DN responds to nonlinear shear deformation by the reversible breaking of the sacrificial bonds, akin to self-healing. Importantly, the recovery under weakly nonlinear deformation yields a steady-state modulus at long times with a value nearly triple the original one, providing a way to tailor the mechanics and topological organization of junctions in such materials. For larger molar mass, a decrease of the Young’s modulus and increase of the strain at break were observed, resulting in an increase of total resilience modulus. Future studies will focus on understanding the microscopic presence and reformation of metal coordination bonds upon shear and recovery. Alternate studies will examine the further increase in the molar mass and, using the protocols presented in this work, assess the role of crystallinity on the mechanical properties of the emerging networks. Finally, the observation of a temperature-dependent modulus is paving the way for the creation of sustainable recyclable materials. It also signifies how iron–catecholate bonding is such an outstanding mechanical strategy in mussels that live at ∼19 °C, a temperature that promotes spontaneous and exothermic iron harvesting from l-3,4-dihydroxyphenylalanine, creating mechanically robust structures.

## Supporting Information Available

NMR spectra of network bonds; analysis of extent of cross-linking reaction; differential scanning calorimetry of SN-1000; small- and wide-angle X-ray scattering; additional linear viscoelastic data of CAT-500 and CAT-1000; control experiments on SN-1000; neo-Hookean and Mooney–Rivlin model fits of SER data; DN-1000 loss tangent versus ω; strain sweep and recovery at 65 °C; Raman shifts before and after break and recovery; and analysis of network properties (PDF)
