# Supplementary material for: Mechanical Properties of Epoxy Networks with Metal Coordination Bonds: Insights from Temperature and Molar Mass Variation
Source: Macromolecules. 2024 Sep 16;57(19):9088–96. doi: 10.1021/acs.macromol.4c01143 (PMC11468226; doi:10.1021/acs.macromol.4c01143)
Supplement: Supplementary file 1 — ma4c01143_si_001.pdf [file ma4c01143_si_001.pdf]

Supplementary Information

Mechanical properties of epoxy networks with metal coordination bonds: insights from temperature and molar mass variation

Benke Li[1], Stelios Alexandris[1], Christos Pantazidis[2], Esmaeel Moghimi[1], Georgios Sakellariou[2], Dimitris Vlassopoulos[1,3], Emmanouela Filippidi[1,3*]

1. Institute of Electronic Structure and Laser, FORTH, Heraklion, 70013, Greece

2. Department of Chemistry, National and Kapodistrian University of Athens, 15784, Greece

3. Department of Materials Science and Engineering, University of Crete, Heraklion, 70013, Greece

ORCID

B. Li	0000-0002-8830-0563
S. Alexandris	0000-0001-7789-7520
C. Pantazidis	0000-0002-1852-2558
E. Moghimi	0000-0001-7268-2715
G. Sakellariou	0000-0003-2329-8084
D. Vlassopoulos	0000-0003-0866-1930
E. Filippidi	0000-0002-4044-0022

Email: filippidi@materials.uoc.gr

The SAXS measurements were performed under the support of the Hellenic Foundation for Research and Technology (H.F.R.I.) Project Number: HFRI- FM17C3-3401, SAXS-SOFT.

Contents:

I. NMR spectra of network bonds (Fig. S1)

II. Analysis of extent of crosslinking reaction (Figs. S2, S3)

III. Differential scanning calorimetry of SN-1000 (Fig. S4)

IV. Small- and wide-angle X-ray scattering (Fig. S5)

V. Additional linear viscoelastic data (CAT-500 and CAT-1000) (Fig. S6)

VI. Iron treatment of CAT-1000 specimens (Fig. S7)

VII. Control experiment: low pH and iron treatment of SN-1000 (Fig. S8)

VIII. Neo-Hookean and Mooney-Rivlin model fits of extensional data (Fig. S9)

IX. DN-1000 loss tangent tan(δ) versus frequency and van Gurp-Palmen plot (Fig. S10)

X. DN-1000 strain sweep and recovery at 65℃ and at 30℃ with delayed oscillatory probing (Figs. S11, S12)

XI. Raman shifts at pH 7.5 before and after break and recovery (Fig. S13).

XII. Analysis of network properties (Tables S1, S2)

I. NMR spectra of network bonds

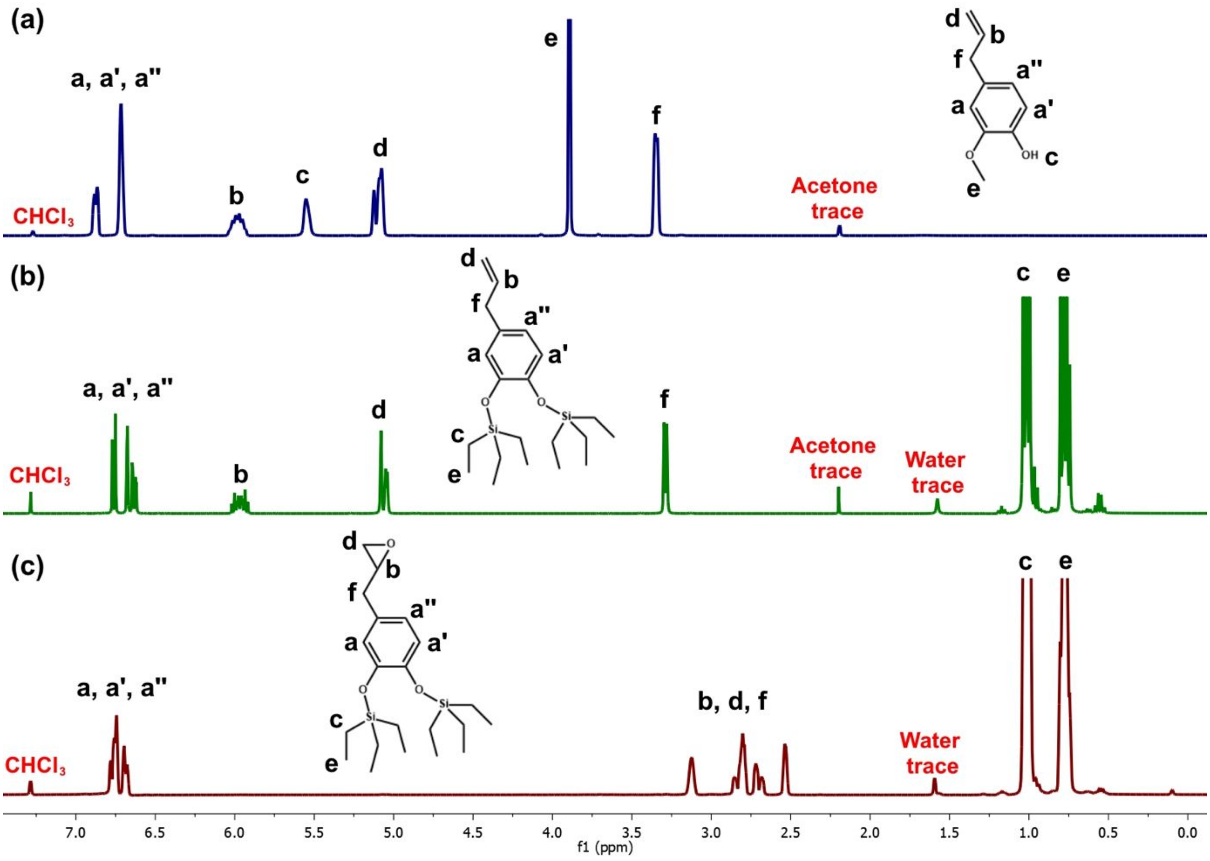

Figure S1. NMR spectra of (a) triethylsilane-protected catechol and (b) triethylsilane-protected catechol epoxide.

II. Analysis of the extent of crosslinking reaction

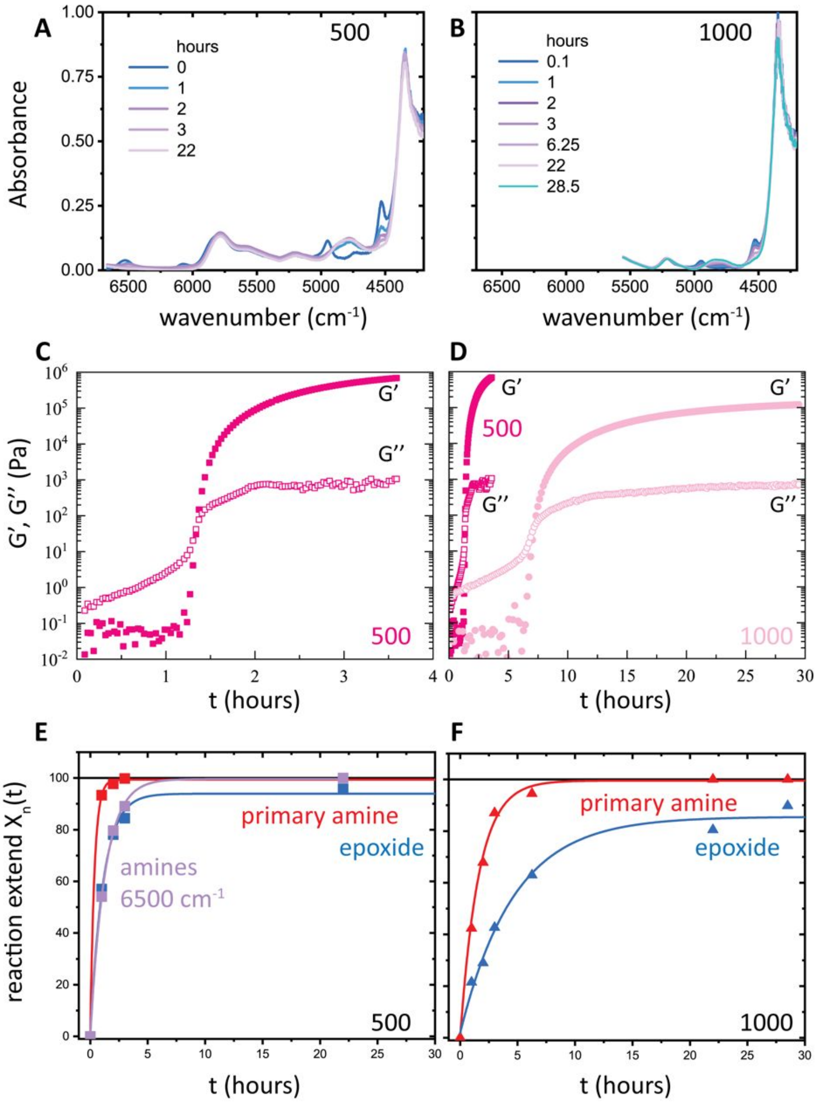

Figure S2. Full IR spectra (A, B), linear rheology at a fixed frequency and amplitude (G', G'') during isothermal cross-linking at 60°C as function of time (C, D). Calculated (points) and fitted (line) reaction extents (E, F) of primary amine (red, ~4950 cm^{-1}), epoxide (blue, ~4525 cm^{-1}) and a combination of the primary and secondary amines (purple, ~6520 cm^{-1}). Data shown for SN-500 (left column) and SN-1000 (right column) PEDGE-DAB single networks.

Reaction extent $X(t) = [A(t=0) - A(t)] / A(t=0)$ from the integrated relevant peaks is fitted to the asymptotic $X(t) = a - b\,c^t$. Having $X(t)$ and $G^*(t)$ as functions of time, we eliminate the parameter of time, plotting $G^*(X)$ in Fig. 2.

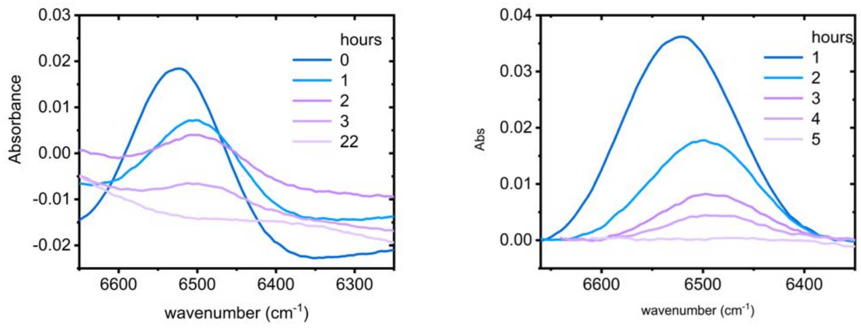

Figure S3. Raw (left) and background-subtracted (right) time-evolution near-IR spectra of the amine peak near 6535 cm^{-1} which corresponds to the combination of primary and secondary amine consumption during the isothermal curing at 60°C. This more complex overtone peak is less informative, as there is overlap of the overtones of both primary and secondary amines corresponding to a shift in lower wavenumbers. The increase at lower wavenumbers is not as pronounced as expected [19].

III. Differential scanning calorimetry of SN-1000

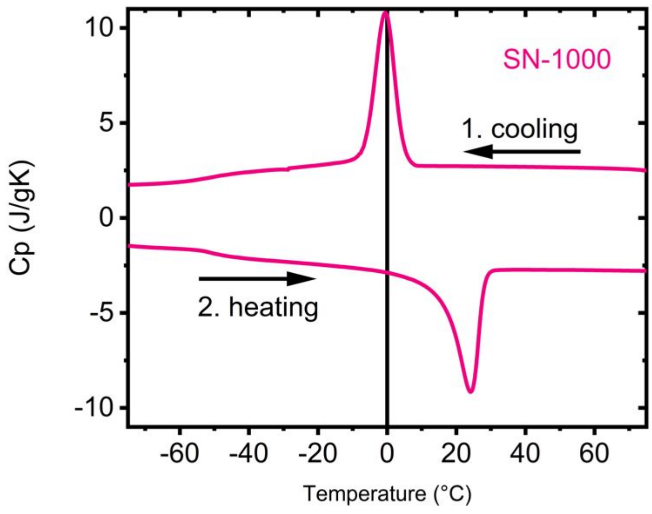

Figure S4. Differential scanning calorimetry curves. Cooling precedes heating. At the rate of 10°C/min, 30°C falls at the end of the melting peak.

IV. Small- and wide-angle X-ray scattering

X-ray scattering measurements were performed on a Xeuss 3.0 system (Xenocs). The X-ray from GeniX3D Cu source (λ = 1.54 Å) was collimated by two pairs of slits. A Dectris Eiger 1M detector was used beamstopless to collect the transmitted and scattered X-ray. Two sample-to-detector distances were used to cover a large scattering wave vector q range.

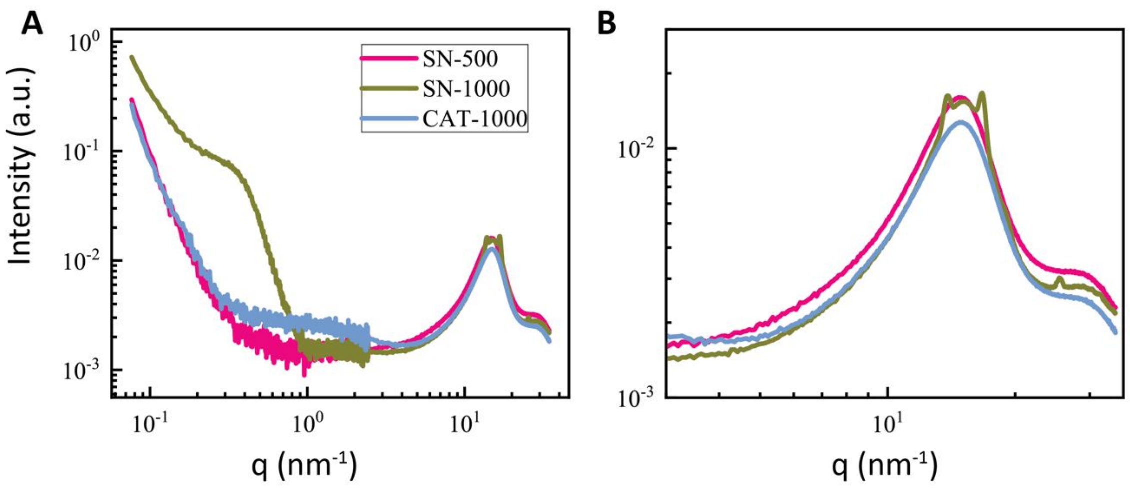

Figure S5. (A) SAXS and WAXS and (B) WAXS at 30°C for the SN-500, SN-1000 and CAT-1000 networks. SN-500 (pink) exhibits lack of crystallinity, has an amorphous PEO peak, and no appreciable heterogeneities in the range of 1 to 20 nm. Thus, any heterogeneities are to be found at larger scales. At those scales heterogeneities can be attributed to inhomogeneous mixing of the precursor solution. SN-1000 (olive) exhibits characteristic PEO crystalline peaks at 1.38 and 1.67 nm^{-1} superimposed on the amorphous peak, which at the CAT-1000 sample (blue) are suppressed, in agreement with DSC traces. The SN-1000 exhibits a high-intensity, broad shoulder at low-q that persists up to 1 nm^{-1} values and is directly linked to the presence of weak crystallinity, via the presence of lamellae of different orientations in a sea of amorphous material. As temperature is increased above 65°C, both the WAXS peaks and the

broad low-q shoulder disappear and slowly reform upon return to room temperature (data not shown). Finally, at the loosely crosslinked CAT-1000 network, a weak broad peak centered at approximately 6.3 nm appears (10 nm^{-1}) suggestive of some possible heterogeneities due to the hydrophobic protected catechol aggregation. However, the sample is swollen for deprotection and iron introduction in downstream processing, so such heterogeneities should not persist in subsequent steps of network treatment.

V. Additional linear viscoelastic data (CAT-500 and CAT-1000)

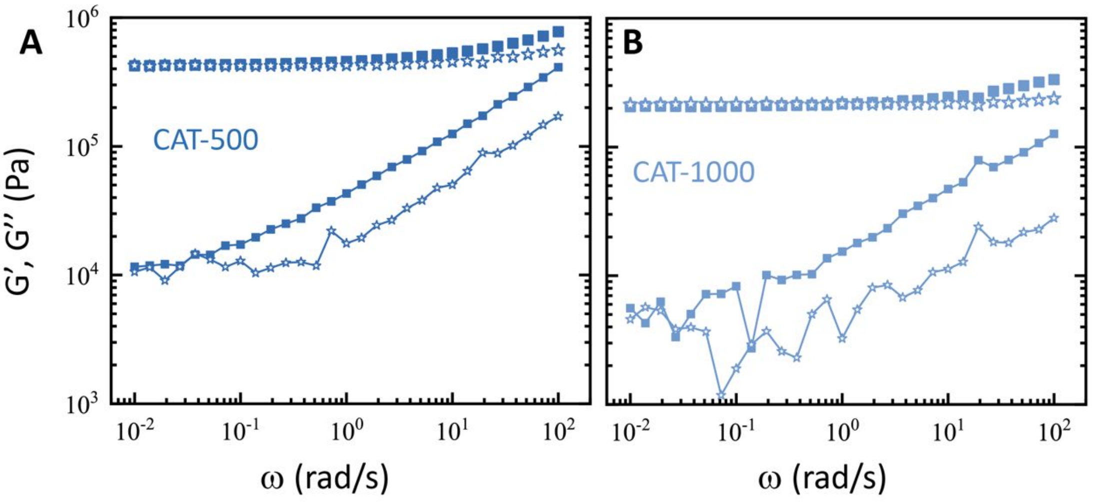

Figure S6. Frequency-dependent storage (G') and loss (G'') moduli at 30°C (filled squares) and 65°C (open stars) for the protected catechol (CAT) networks synthesized from (A) 500 g/mol and (B) 1000 g/mol PEGDE.

VI. Iron treatment of CAT-1000 specimens

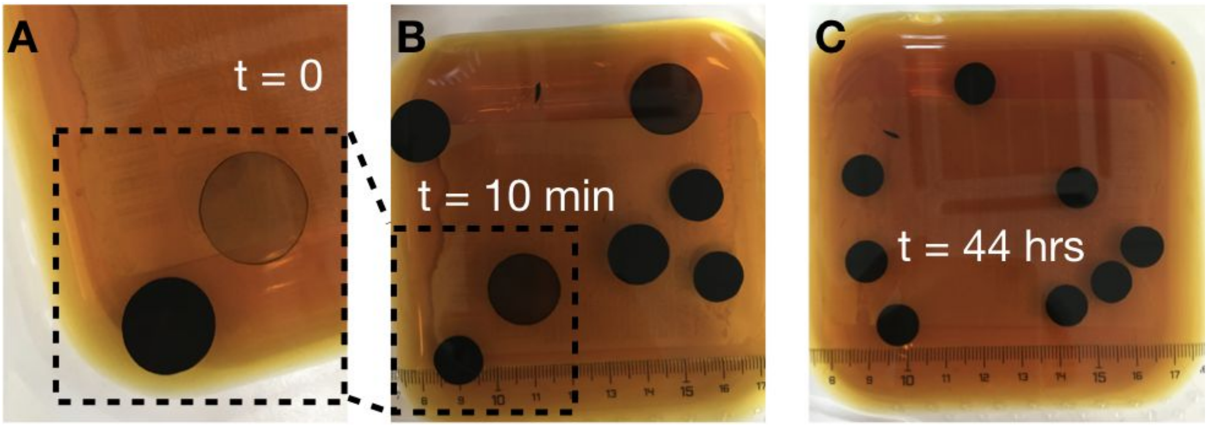

Figure S7. (A) Introduction of CAT-1000 specimens directly after a 24 hour HCl, pH 2.5 deprotection step into the iron/bicine solution. At t = 0, the new transparent specimen is coming in contact with the iron/bicine solution. (B) 10 min later iron has visibly diffused inside. A few minutes later the sample will start visibly shrinking. (C) Typically, after 24 hrs (here shown after 44 hours) specimens have attained a final reduced size from 19.25 mm to 11 mm. Systematic studies on the effect of bicine concentration need to be performed in the future.

VII. Control experiment: low pH and iron treatment of SN-1000

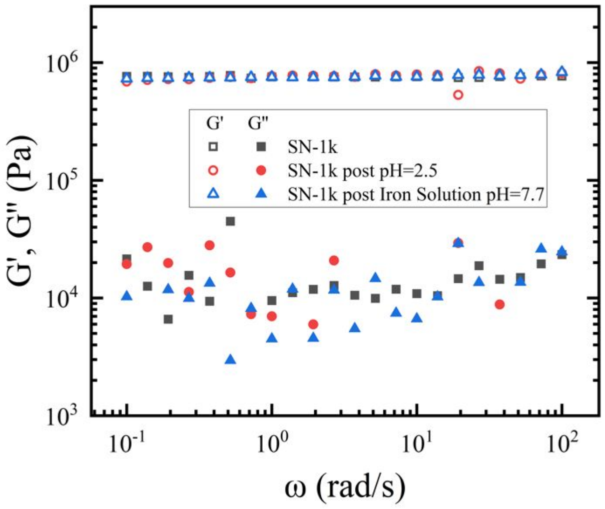

Figure S8. Control experiment. Overnight SN-1000 treatment at HCl solution, pH 2.5 which results in swelling, offering an opportunity for unreacted chains to diffuse out of the network

followed by re-drying and rheology measurement. Mimicking the CAT-1k iron treatment, swollen SN-1000 in HCl were directly introduced to the iron/bicine solution at pH 7.7, dried, followed by the rheology measurement. No evidence of altered mechanics as measured by G' and G" is found, indicative of no unreacted chains diffusing out of the network, and no PEG oxygens linked via iron atoms, acting as cross-links.

VIII. Neo-Hookean and Mooney-Rivlin fits of extensional data

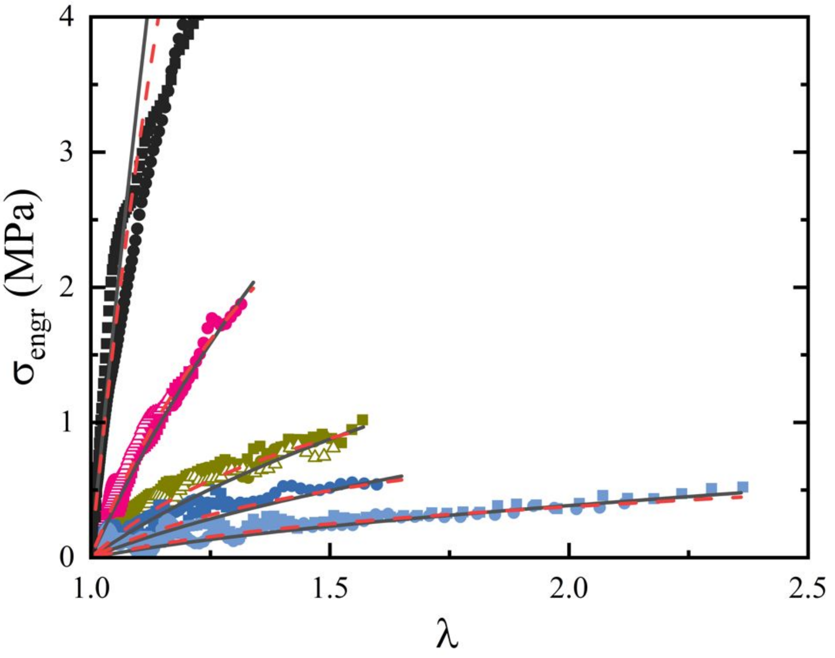

Figure S9. Fits of SER extensional data with the Neo-Hookean model (black solid lines) and the Mooney-Rivlin (red dashed) models.

IX. DN-1000 loss tangent tan(δ) versus frequency and van Gurp-Palmen plot

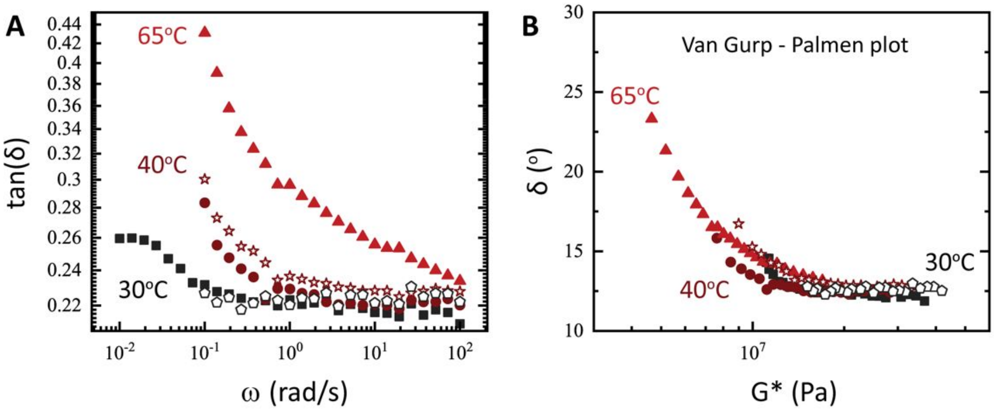

Figure S10. For the dual network DN-1000 (A) loss tangent tan(δ) versus frequency and (B) van Gurp-Palmen plot. Temperature was increased from 30°C to 40°C to 65°C (filled symbols) and subsequently decreased back to 40°C and 30°C (open symbols).

X. Fe-treated network (DN-1000) strain sweep and recovery at 65°C and at 30°C with delayed oscillatory probing

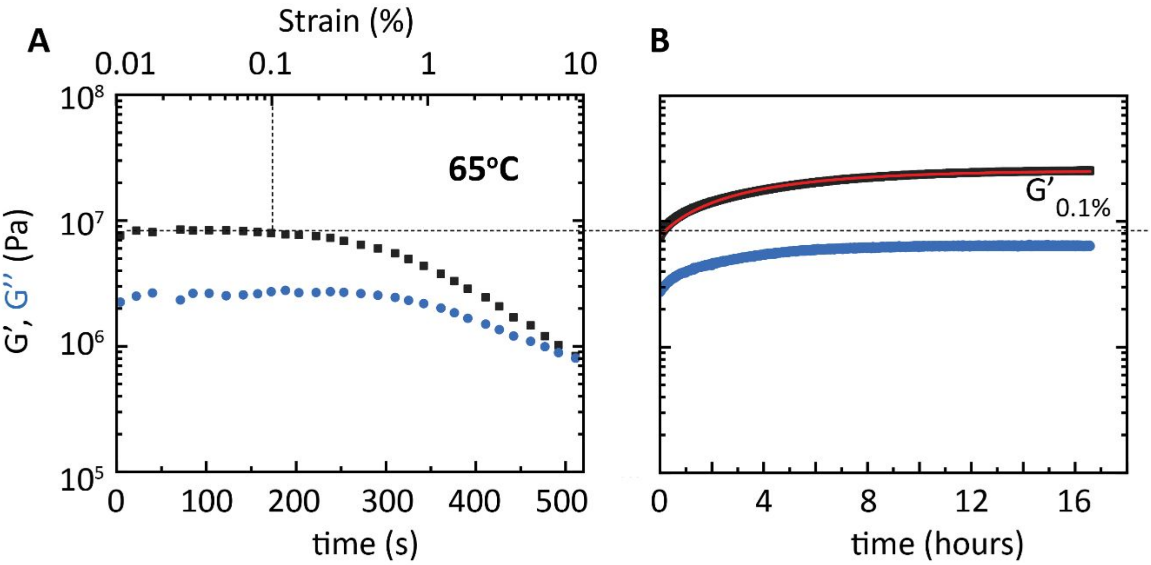

Figure S11. Strain sweep and recovery of DN-1000 network at 65°C. (A) A dynamic shear strain sweep in the range 0.01 - 10% partially fractures the sample. (B) Time-dependent

recovery at a frequency $\omega = 1$ rad/s and strain amplitude $\gamma = 0.1\%$. The dashed line shows initial modulus value at strain 0.1%. Red is fit to $G'(t) = (G'_{\infty} - G'_0)\left[1 - e^{-\left(t/\tau_{net}\right)^{\beta}}\right] + G'_0$. $\tau_{net} = 4.5$ hours is the network's rebuilding time, fitting parameter $\beta = 1.002$ is the stretch parameter.

Before fracture, $G'_{0.1\%,pre} = 7.63\ MPa$, whereas the plateau modulus after recovery is $G'_{0.1\%,post} = 25.7\ MPa$, a 3.4–fold increase. The persistence of the effect at 65°C, a temperature well above PEGDE's crystallization and melting is excluding the possibility of G' increase due to shear-induced crystallization.

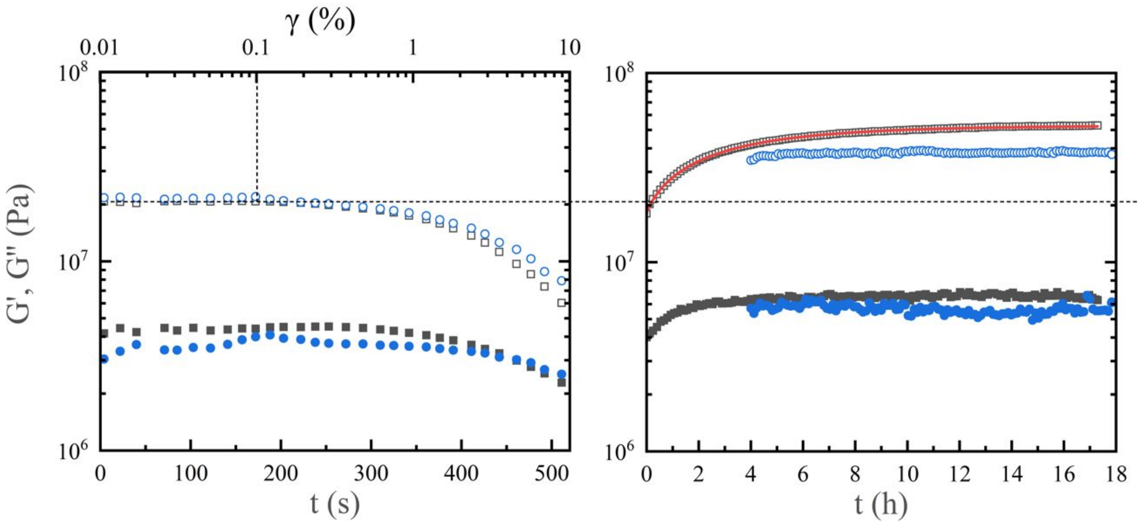

Figure S12. Strain sweep and recovery of DN-1000 network at 30°C with a 4-hr delay in oscillatory probing. In this control experiment, we stain the sample as above (Fig. S10), but delay the oscillation, in order to exclude the possibility of the cyclic linear oscillation introducing an artifact to the recovery. It is evident that the recovery above the original G' perrsists and is within experimental sample variation.

XI. Raman shifts at pH 7.5 before and after break and recovery

Raman spectroscopy was performed with a 532 nm laser on cross-sections of samples with the DXR3xi Raman microscope by Thermo Scientific.

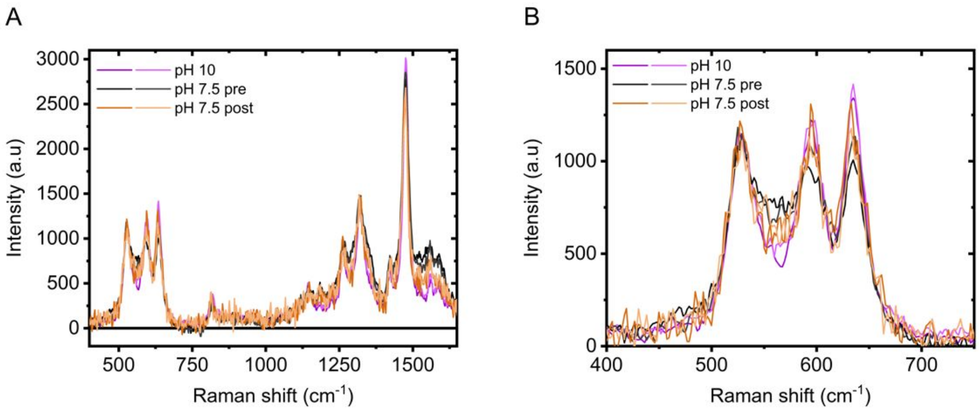

Figure S13. Raman shifts at room temperature of DN-1000 treated at pH 10 (pink), or at pH 7.5 before (black) and after strain and recovery (orange). No conclusions can be drawn, as the spectra appear similar. Therefore, either the spatial heterogeneity is larger than the differences among samples, or/and the current resolution ability is not optimal.

XII. Analysis of network properties

Table S1. Theoretical prediction of masses and mol quantities if all catechols participate in all tris- coordinate binding with Fe^{3+}.

	PEGDE 1k	DAB	deprotected and Fe-bound CAT	Fe^{3+}	tris complex
MW (g/mol)	1000	88.15	164.16	55.845	548.325
mol % before Fe(III) introduction	29.2	25.8	45	—	—
moles after	29.2	25.8	45	45/3 = 15	15

Fe(III) introduction					
mol % after Fe(III) introduction	41.71	36.86	—	—	21.43
mass (g)	29200	2274.27	7387.2	837.675	8224.875
total mass (g)	39699.145				8224.875
our sample (mg)	7.4				
our sample composition (mg)	5.443	0.424	1.377	0.156	1.533
mass % composition	73.55	5.73	—	—	20.72

Table S2. Results from the DFT calculations of Reference [21] for the Gibbs free energy of tris-catecholate – Fe(III) complex formation (ΔG).

Temperature (K)	Temperature (^{o}C)	ΔG (kJ/mol of complex)	ΔG (J/g of complex)
298.15	25	- 26.583	- 48.48
340	66.85	- 20.181	-36.80

$\Delta(\Delta G_{66.85}{}^{o}{}_{C} - \Delta G_{25}{}^{o}{}_{C}) = -36.80 + 48.48$ J/g complex = + 11.68 J/g complex.

DFT estimate for our actual sample of 7.4 mg which contains 1.533 mg of complex:

ΔG (J/g of complex) x mass available complex = 11.68 J/g complex x 1.533 10^{-3} g complex

= 17.9 mJ or 17.9 mJ / 7.4 mg sample = 2420 J/kg of sample.

The sample was a disk of radius 2 mm and height 0.44 mm, with macroscopic volume

$V_{sample} = 5.53 \times 10^{-9}$ m^3, thus approximate density 1338 kg/m^3.

Therefore, 2420 J/kg sample x 1338 kg/m^3 sample = 3.239 10^6 J/m^3 = 3.239 10^{-21} J/nm^3, or 0.75 k_BT / nm^3 at a median temperature of 40℃.

This is the estimated average reduction in Gibbs free energy per volume, or equivalently a reduction in the network modulus. The linear viscoelastic data indicate a reduction in the plateau modulus from 30℃ to 65℃ of 9.2 MPa.

9.2 MPa = 9.2 ·10^6 J / m^3 = 9.2 · 10^6 J / 10^{27} nm^3 = 9.2 · 10^{-21} J/nm^3 which is only 2.84 times higher energy density compared to the gas phase DFT estimation.
